# Negative Pressure Wound Therapy and Traditional Dressing: An Italian Health Technology Assessment Evaluation

**DOI:** 10.3390/ijerph20032400

**Published:** 2023-01-29

**Authors:** Dora Nicolazzo, Elena Rusin, Alessandra Varese, Margherita Galassi

**Affiliations:** 1 IIG Philips, 20126 Milan, Italy; 2 Ab Medica Spa, 20023 Cerro Maggiore, Italy; 3 A.O.U Città dalla Salute e della Scienza di Torino, 10126 Torino, Italy; 4 Istituto Nazionale per lo Studio e la Cura dei Tumori, 20133 Milan, Italy

**Keywords:** NPWT, negative pressure wound therapy, traditional dressing, PAD, peripheral arterial disease

## Abstract

This evaluation shows the main advantages related to the introduction of negative pressure wound therapy (NPWT) in Italian clinical practice for the management of incisions in vascular surgery in patients suffering from peripheral arterial disease (PAD) and at risk of postoperative complications, compared to treatment with traditional dressings. A health technology assessment (HTA) activity was conducted assuming the hospital perspective, within a 12-month time horizon. The nine EUnetHTA Core Model dimensions were deeply explored, using scientific evidence on the topic, real-life data, and healthcare professionals’ perceptions. The evaluation shows that the use of NPWT has had a positive impact in terms of higher clinical effectiveness and safety profile. The process mapping highlights how NPWT allows a reduction of 2.5 hospitalization days compared with standard dressing, with the consequent benefits considering economic, organizational, and social aspects. A significant economic saving per patient emerged, with an overall optimization of the patient’s clinical pathway, impacting positively on the hospital’s capacity. The budget impact analysis shows that the higher number of patients treated with NPWT, the higher the economic advantages. Furthermore, assuming the patient’s perspective, it would generate an overall reduction in social costs of 28%. In conclusion, the results of this study provide helpful evidence-based information to policymakers through examinations of the relative values of intervention, thus supporting the overall hospital and institutional decision-making process to define appropriate areas of investments, leading to the achievement of not only higher clinical outcomes, but also important social, economic, and organizational advantages.

## 1. Introduction

Surgical site infections (SSIs) are one of the most frequent healthcare-associated infections (HAI) and are considered the most frequent complication in surgical patients, being responsible for 38% of all infections (WHO 2011 report on the burden of endemic healthcare-associated infection worldwide).

The incidence of SSIs varies according to different factors (i.e., age, type of surgery, and surgical incision classification), with an incidence rate ranging from 0.5% to 10.1%, depending on the type of surgical procedure (Annual Epidemiological Report on Communicable Diseases in Europe, 2017 [1]). SSIs are frequently associated with several repercussions that negatively impact patients’ outcomes such as, for example: long post-surgery in-hospital stays, additional surgical procedures, and a high mortality rate.

The literature available on the topic confirms that SSIs represent a significant burden to patients and healthcare systems in terms of length of stays (LOS), time spent in an ICU (intensive care unit), readmission to hospital, long-term disability, contribution to the spread of antibiotic resistance, substantial financial burden, and high costs for patients and families.

This enormous burden of morbidity and mortality has led the public, regulatory agencies, and payers to focus on ways to reduce the consequences and costs of these infections. As a result, SSIs and, more specifically, hospitals’ performances related to the prevention of SSIs are subject to public reporting requirements in many countries. For all the above reasons, the surveillance of SSIs is a crucial topic for the healthcare system, and the need to access the best therapy or strategy available to control and reduce its incidence is essential.

Every surgical procedure has its own set of risks for SSIs. One of the diseases with the highest rate of SSI is peripheral artery disease (PAD) which is estimated to affect 200 million people worldwide [2].

This disease is progressive, affects the peripheral circulatory system, and is characterized by a reduced blood flow to the arterial vessels of the lower limbs and upper limbs. PAD is also associated with a reduced functional capacity and increased mortality and risk of cardiovascular morbidity; if classified as severe, it requires the patient to undergo surgery such as angioplasty, bypass, or amputation. According to the study by Vogel and colleagues [3], this type of surgery has one of the highest rates of SSI, delaying healing and causing high morbidity, mortality, as well as generating a significant economic and social burden.

According to the study conducted by Shu et al. [2], the main risk factors are related to advanced age, hypertension, dyslipidemia, and diabetes. In fact, 20% to 30% of the population affected by PAD suffers from diabetes, and diabetic patients are four to five times more likely to develop this disease. Other risk factors include smoking: smokers are two times more likely to develop PAD [4].

Patients suffering from severe PAD undergo surgery such as angioplasty, bypass, or amputation, thus being interventions related to a higher rate of SSIs [3]: in particular, according to the Italian Society of Angiology and Vascular Pathology, the population of patients over 60 years old affected by this disease in Italy is equal to 20%.

Within clinical practice, there are different technologies dedicated to the treatment of surgical wounds. In this view, it emerged that wound management involves a comprehensive care plan, considering all factors contributing to and affecting the wound and the patient.

The standard of care for the management of surgical wounds, thus preventing the SSI occurrence rate, is represented by a traditional dressing or advanced wound care. On the one hand, a traditional dressing is identified as a material placed in direct contact with the sole function of hemostasis, coverage, and protection, with a purpose of maintaining a humid microenvironment and constant temperature, removing exudates and necrotic material, protecting from exogenous infections, being permeable to oxygen, and reducing trauma to change. On the other hand, among the different types of advanced technologies used to reduce the risk of SSIs, there has been much interest in the use of negative pressure wound therapy (NPWT). Evidence suggests that NPWT may accelerate healing times, reduce both the length of hospital stay and the frequency of dressing changes, and improve patients’ quality of life. NPWT has thus become a viable wound care option since its introduction two decades ago. For many different surgeries, such as general, abdominal, vascular, and orthopedics surgery, NPWT is an integral adjunct treatment to enhance different interventions in the surgical pathway and surgeons have discovered that foam-based negative pressure dressings applied over closed incisions can also be beneficial in preventing incision complications. Specifically, it consists of an open-cell polyurethane foam to ensure the optimal distribution of negative pressure across the incision surface. Thus, the NPWT system is designed to manage surgical incisions and surround intact skin in patients considered at high-risk of developing complications after surgery. It consists of an open-cell polyurethane foam to ensure the optimal distribution of negative pressure across the incision surface [5], which can be used within both the hospital and the homecare setting. One of the most relevant differences between a traditional dressing and advanced wound care lies mainly in a lower frequency of replacement and especially in the reduction of healing time which are certainly not insignificant advantages both for the patients and for the healthcare providers.

Focusing on the use of such a treatment strategy within vascular surgery, NPWT protects the incision from external contamination, helps to hold incision edges together, removes fluid and infection materials, and delivers continuous negative pressure at −125 mmHg for up to seven days. As such, NPWT utilization in PAD may improve wound healing by providing moist wound conditions, reducing exudate, controlling wound-bed infection, and stimulating granulation. Furthermore, home-based NPWT allows the patient to recover in the community while minimizing risks of prolonged hospitalization [6].

Despite the relevance of the topic, no consensus exists in the clinical practice concerning the routine use of NPWT as a strategy to prevent SSIs. Moving on from these premises, the present paper aims at defining the main advantages related to the introduction of NPWT in comparison to traditional/advanced dressings in the management of inguinal incisions, thus evaluating the potential reduction in postoperative infections within the field of vascular and endoprosthetic surgery in the Italian setting. The achievement of the aforementioned objective would attempt to answer the following policy question: “Which is the added value in implementing advanced wound care in the clinical practice, enhancing not only a higher clinical outcome, but also an organizational and economic hospital sustainability?”

## 2. Materials and Methods

A health technology assessment (HTA) analysis has been implemented in Italy with the main objective of comparing the introduction, into clinical practice, of the innovative NPWT technology with the already well-known traditional dressing.

The analysis assumed the hospital perspective and considered a 12-month time horizon. To better evaluate the results and demonstrate the true value of the device in reducing the after-surgery infection rate, the study has been restricted to the management of an inguinal incision in the field of vascular and endoprosthetic surgery inside an Italian hospital in Piedmont (Northern Italy).

The assessment was conducted, examining the following dimensions as suggested by the EunetHTA Core Model [7]: (i) general relevance; (ii) description of the technologies; (iii) safety; (iv) efficacy; (v) economic and financial impact; (vi) equity aspects; (vii) legal implications; (viii) social and ethical factors; and ix) organizational issues.

For the deployment of the above dimensions, both qualitative and quantitative methods were used: (i) a systematic literature review for the definition of the comparative efficacy and safety data; (ii) administration of qualitative questionnaires, completed by three healthcare professionals (according to a 7-item Likert scale, ranging from −3 to +3 [8]; and (iii) health economics tools, useful for the economic evaluation of the clinical pathway and budget impact analysis, and for the definition of the organizational advantages, in terms of time savings.

Before starting the assessment of the dimensions, a PICO approach (Problem/population, Intervention, Comparator, and Outcome) for the literature validation has been identified to fine-tune the research question. The following PICO was discussed and proposed.

***P (population):*** Patients over 60 affected by PAD who have undergone hospitalization, for vascular surgery for lower limb revascularization.

***I (intervention):*** NPWT, negative pressure wound therapy.

***C (comparator):*** Traditional dressing.

***O (outcome):*** Surgical site infection rate (SSI), length of stay (LOS), and re-hospitalization rate.

The literature evidence strategy was conducted through a search on literature databases (Pubmed, Embase, and Cochrane Library), using the following keywords: “NPWT”, “negative pressure wound therapy”, “Groin & Vascular”, “traditional dressing”, “surgical site infection rate”, “length of stay”, and “re-hospitalization rate”.

No time or language limits were utilized. Only papers focusing on the investigated NPWT within the vascular and endoprosthetic settings were included in the analysis, thus preferring a study design presenting a head-to-head comparison between NPWT and traditional dressings.

The validation of the scientific evidence available on the topic was performed through the Critical Appraisal Skills Program (CASP) checklist, thus defining the overall quality of the records included.

The literature has been used to highlight the efficacy profile of negative pressure in terms of reducing the rate of surgical site infections and consequently decreasing the risk of rehospitalization, re-operation, and mortality. All these factors impose a significant increase in additional organizational efforts and costs for hospitals.

Another data source used for examining the HTA dimensions consists in the healthcare professional’s perceptions retrieval. A qualitative questionnaire was administered to three healthcare professionals with skills and knowledge on both NPWT and traditional dressings. The collection of their perceptions was utilized to deploy the efficacy and safety profiles, but also the equity, social, legal, and organizational aspects, considering a comparative approach between traditional dressings and NPWT in accordance with a 7-item Likert scale, ranging from −3 to +3 [8], based on the specific items derived from EUnetHTA Core Model [7].

Focusing on the quantitative assessment of both the economic and organizational dimensions, at first a process mapping technique and an activity-based costing approach were implemented to define the costs directly sustained by hospitals in the management of PAD patients after surgery. The following hospital costs, representing the input data of the economic evaluation, were considered, based on both real-life data and the literature evidence: (i) the human resources involved, in terms of assistance time (measured as minutes) spent by the nurse, the clinician, or any other healthcare professionals during the entire management of the patient undergoing surgery; (ii) laboratory and radiologic exams, in terms of the typology and quantity of procedures delivered to the patient, based on the length of stay; (iii) traditional dressings or NPWT, thus considering all the consumables utilized; (iv) drugs and any other medications administered to patients during surgery or hospitalization. Only the direct costs were accordingly investigated, and the total cost for each patient was calculated by multiplying the quantity of resources consumed by their unit cost. In addition, general and fixed hospital costs were integrated, consisting of all those costs different from labor factors, consumables, and equipment usage, which was necessary to taking charge patients because they provide the logistic and infrastructure support, in the measure of 20% of the direct costs [9].

Based on the above, the following hypotheses were assumed.

- Concerning the assistance time within the hospitalization, an estimated 120 min per day of assistance was considered for the ward nurse, 10 min per day for the ward physician, and 60 min per day for any other healthcare professionals. The costs of both traditional dressings and NPWT were derived from the hospital acquisition costs, based on the most recent specific tender procedures.
- The length of stay was derived from the literature evidence available on the topic. Lee and colleagues [10] reported a length of stay equal to 6.5 days for patients to whom NPWT is applied in the operating room, and equal to 9 days for patients treated with traditional dressings.
- The dressing will be changed exclusively at day 7 by removing the NPWT and applying a traditional dressing, compared to the daily dressings required by the traditional pathway (nine changes based on the length of stay).
- The application of the NPWT allows for a reduction in outpatient follow-ups for dressing changes, being only one in the delivery of the innovative treatment and two with traditional dressings.

**Figure 1:**
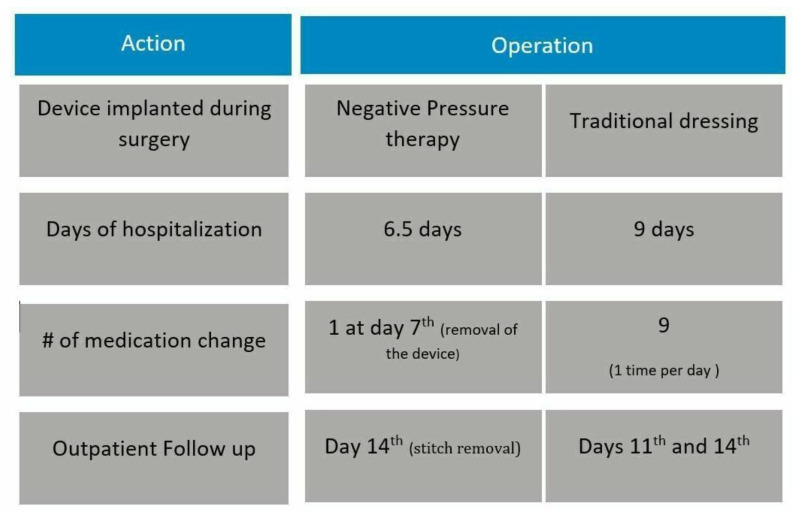
Patient pathways considering the innovative or traditional technology.

Figure 1 describes both patient pathways, according to the type of medical device used for the treatment of the wound (conventional dressings vs. NPWT).

After the definition of the costs related to the management of patients based on either NPWT or traditional dressings, a budget impact analysis (BIA) was performed to define the economic sustainability of NPWT adoption on the hospital budget, assuming a 12-month time horizon and considering the number of admissions performed within a medium-size hospital for lower limb revascularization surgery with groin injury (N = 811 patients/year). The budget impact analysis includes the economic evaluation of the potential development of a surgical site infection, as well as re-operation or re-hospitalization [10,11,12]. On the one hand, SSIs may occur in 21.60% or 10.10% of patients treated with traditional dressing or NPWT, respectively. This complication would imply the administration of antibiotics, as well as a prolongation of the overall length of stay, quantified in 9.7 additional days. On the other hand, 16.7% and 6.8% of patients undergoing traditional dressings or NPWT, respectively, would require additional re-hospitalization within 30 days from discharge. In addition, the BIA also comprised the portion of patients for whom a second surgery is needed, equal to 18.30% in the traditional dressing arm and 8.5% in the NPWT arm.

An additional quantitative analysis was performed at the organizational level, to define the potential capability of NPWT to save hospital beds, given the reduction in the hospital length of stay. In particular, the analysis hypothesized a complete technological replacement rate, thus comparing the baseline scenario in which all patients are treated with conventional dressings, to an innovative and best-case scenario where NPWT is applied to all the surgical PAD patients.

## 3. Results

### 3.1. Literature Review

**Figure 2:**
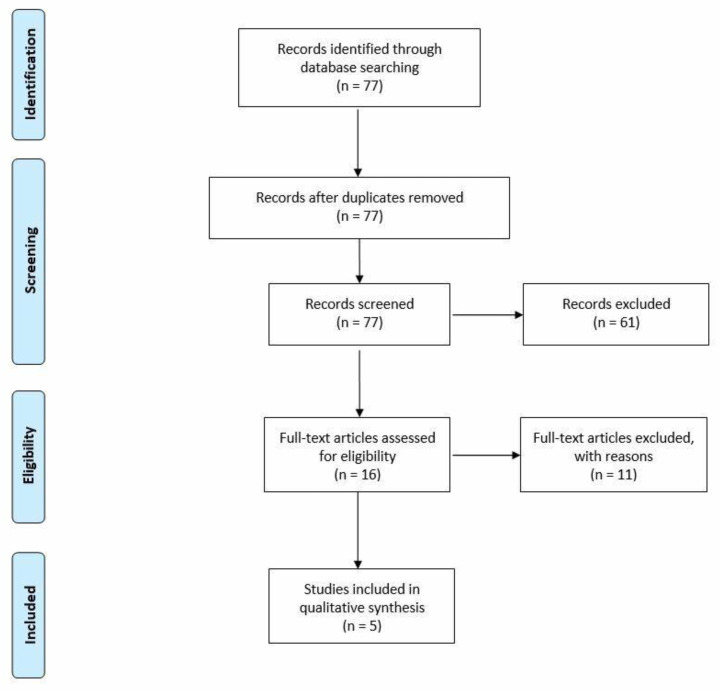
Prisma flowchart of the literature review conducted.

The PRISMA Flow Diagram reported in Figure 2 depicts that out of the 77 records, only 5 studies were included in the analysis because of their focus on the topic under assessment [10,11,12,13,14].The CASP checklist demonstrates the quality of the results, which were considered complete and replicable.

In general terms, one of the most relevant differences between the two dressings under consideration lies mainly in a lower frequency of substitution and above all in the reduction of healing time, which are certainly not insignificant advantages both for the patient and for the healthcare company providing the service.

The endpoints used for the safety dimension are re-hospitalization and re-operation rates; otherwise, the efficacy indicator used is represented by the SSI rate.

Focusing on the safety dimension, the rates of re-hospitalization and re-operation due to complications at 30 days were identified and extrapolated from Kwon and colleagues [10,11], revealing the NPWT significantly represents the safest solution. The NPWT decreased both rates by about 10 percentage points compared to the traditional dressing (re-operation: 18.3% traditional dressing vs. 8.5% NPWT; re-hospitalization: 16.7% traditional dressing vs. 6.8% NPWT).

As already mentioned, wounds from vascular surgery have among the highest rates of SSIs. A high rate of infections means, from a hospital perspective, a higher risk of prolonged hospital stays, and an increased likelihood of re-hospitalization, re-operation, and mortality. These additional risks are all factors that impose significant additional costs on the hospital setting. As shown in Table 1, in all five studies considered, the rate of SSI is significantly lower in patients treated with NPWT and, unlike the other parameters considered, is always significant with *p*-values less than 0.05.

### 3.2. Quantitative Results: Economic and Organizational Assessment

The economic analysis, reported in Table 2, shows that the cost that mainly affects the overall hospital resources absorption is related to the hospitalization phase (EUR 5250 for NPWT vs. EUR 7280 for traditional dressings). In fact, with the use of NPWT, there is a reduction of 2.5 days of post-surgery hospitalization equal to a cost reduction of EUR 2030 and also a reduction in costs and time of human resources involved.

Based on the above, the routine implementation of NPWT would lead to an economic saving per patient equal to 15% (EUR 1722).

Taking into consideration the occurrence of adverse events and their incidence rates quantified in the reference studies, it is noted that they are lower with NPWT: this reduction generates a decrease in costs related to the management and resolution of complications. According to De Lissovoy’s study [15], on average, an SSI increases the hospital stay by 9.7 days with an additional cost of EUR 5820 (assuming that the cost of a day of hospitalization is equal to EUR 600, in the specific operative ward of reference for the analysis).

Table 3 reports the results from the BIA, according to the comparison among the baseline scenario and four different innovative scenarios. The more the NPWT is implemented in the clinical practice, the higher the economic savings, ranging from a minimum of 1.15% (Scenario 2) to a maximum of 18.56% (Scenario 4).

Indeed, the implementation of NPWT to only 20% of the eligible population did not represent a sustainable solution for hospitals, thus being associated to an increase in costs equal to 2.26%.

It should be noted that the advantage generated by the introduction of NPWT should not be relegated only to the economic sphere, but may also have substantial benefits from an organizational point of view, especially because the innovative technology allows a reduction in the overall length of stay.

Considering a complete replacement rate (thus comparing the base scenario with Scenario 4), the possibility to implement NPWT to all the eligible patients would lead to a reduction in hospitalization days equal to 2027 days (7299 days for traditional dressings vs. 5272 for NPWT). This saving in inpatient days would also result in the ability to treat and care for an additional number of patients (N = 312 patients), thus increasing the overall hospital accessibility to care for patients requiring surgery, potentially solving an urgent problem related to the recovery of not-delivered services and waiting lists, especially impacting elective surgery.

### 3.3. Qualitative Results

Table 4 depicts the qualitative perception of the healthcare professionals involved in the analysis, based on an evaluation scale ranging from −3 to +3. 

From a safety point of view, NPWT may be considered the preferable solution, with an overall average value equal to 0.78 (vs. 0.03 for the traditional dressing). A discriminating factor appears to be the learning curve in both patient selection and the management of adverse events. Especially with reference to this last point, the professionals involved agreed in stating that NPWT is related to a lower occurrence of both severe (NPWT: 1 vs. traditional dressing: −1) and moderate adverse events (NPWT: 1.67 vs. traditional dressing: 0.33).

The same trend emerged regarding the effectiveness profile: NPWT has thus acquired a higher score than the comparator (2.17 vs. 0.58). NPWT presents the ability to activate the stimulation and promotion of the regenerative processes (2.33 vs. 0.33), as well as the prevention of infections (2.33 vs. 0.67).

Access to the new technology does not appear to represent an important criticality from the point of view of the equity dimension. The new technology has no impact on the phenomenon of healthcare migration and does not seem to be relevant to the generation of waiting lists. Accessibility could be perceived as a limitation of the new technology, not because of the complexity of its use, but because of the limitations posed by the negative medication machine allocation in the various Italian regions’ healthcare hospitals and providers (−0.33 vs. 0.66).

Focusing on the patients’ perspective, thus examining the social aspects, NPWT could be considered the preferable solution for wound care. NPWT is strictly related to the potential achievement of a better quality of life perceived by both patients (NPWT: 1.33 vs. traditional dressing: 0.33) and caregivers (NPWT: 1.33 vs. traditional dressing: 0.00). In addition, a decrease in social costs could emerge (NPWT: 1.33 vs. traditional dressing: 1.00), given the lower hospitalization rate (NPWT: 1.67 vs. traditional dressing: 0.33)

The two technologies could be considered superimposable for legal aspects. The analysis of this dimension is relevant to guide evaluators in recognizing the relevant legal issues to consider when evaluating the technology and provide proper guidance to decision makers.

## 4. Discussion

The prevalence of concomitant comorbidities, due to the increase in the average age of the population, and the issue of the development of SSIs especially in patients with certain comorbidities should lead to an evolution of healthcare systems in a way that allows them to address the emerging health challenges associated with the respective health and social costs [16]. For example, SSIs of groin incisions are a major source of patient morbidity leading to increased rates of reintervention, hospital length of stay, and even mortality after vascular reconstruction [17].

Within clinical practice, there are different technologies devoted to the treatment of surgical wounds. In addition to traditional dressings, there are more innovative technologies that use negative pressure.

The NPWT system is designed to manage surgical incisions and the surrounding intact skin in patients at risk of post-surgery complications.

Moreover, productivity losses and reduced quality of life due to an adverse medical event have a negative impact on societal and community burden; healthcare sustainable policies, innovative procedures, and new clinical interventions provide opportunities for the reallocation of resources and increase quality of life.

In recent years, treatment with negative pressure dressings has changed the landscape of the management of several complicated skin lesions. The consensus on the use of these innovative dressings, based on solid clinical evidences, is largely consistent across devices [18,19].

The present study partially attempts to cover this knowledge gap by applying a multidimensional assessment and by showing the preferability in using negative pressure dressings compared to traditional dressings; indeed, negative pressure technology has been applied to closed surgical incisions at-risk of infection for about fifteen years and is supported by many studies [20], confirming that its use for surgical incisions reduces the rate of infection at the surgical site. In addition, according to this study, NPWT presents a lower re-hospitalization and re-operation rate than traditional dressings, with consequent organizational and social advantages.

The results have demonstrated that NPWT is considered the preferable solution from an efficacy and safety perspective, thanks to its known activity of stimulation and the promotion of regenerative processes, and has a key role in reducing complications still considered among the most frequent and serious. The same superiority, in terms of safety, emerged from the analysis of the questionnaires submitted to the experts who found themselves in agreement in declaring that treatment with negative pressure is correlated with a lower occurrence of high and medium side effects. This implies significant benefits from the patients’ management. In this view, the economic analysis has demonstrated the capability of NPWT to optimize the overall PAD patients’ clinical pathway, with an overall saving per patient equal to 15%.

In addition, the BIA confirms the economic sustainability of NPWT for the hospital budget. The economic results presented here are in line with scientific evidence on the topic: in high-risk patients and high-risk surgical procedures, ciNPT (closed incision negative pressure) appears to have the potential to reduce surgical incision complications and a surgical cost per patient up to $9000, depending on the type of incision and patient risk factors [21].

The possibility of optimizing the patient’s clinical pathway is particularly due to the capability of NPWT in reducing the length of stay, thus consequently releasing hospital beds with the opportunity to increase the overall accessibility to care. Furthermore, the reduction in hospital stays could have a favorable impact on a decrease in the patients’ productivity losses as well as a faster recovery rate, with a positive social impact. Since NPWT is related to a lower length of stay and requires a lower number of follow-up procedures, it could generate a reduction in the social costs sustained by both the patients and the related caregivers equal to 28% (EUR 710 for traditional dressing vs. EUR 985 for NPWT). The analysis of the social aspect is becoming increasingly important since patients, their families, and caregivers are being recognized as therapeutic area experts. The patients’ associations worldwide have a clear desire to give their contribution, highlighting the value of medical devices by bringing their voice into the HTA discussion. Incorporating patients’ insights may help to improve the benefits and fulfil the unmet need not only during hospital treatment but also in the homecare setting, thus being consistent with the recent National Recovery and Resilience Plan (NRRP), which advocates the possibility of home management, especially in the case of frail patients, and presents the aim of strengthening local prevention and healthcare services, modernizing and digitizing the health system, and ensuring equal access to care.

It should be noted here that the HTA evaluation conducted has taken into consideration the hospital point of view. NPWT is thus a technology that could be easily used within a homecare setting for patients with chronic wounds, being a key factor in improving the self-care ability and confidence of patients. Much can be gleaned from the patient experience, but the future success of portable NPWT will be measured on time in care and therefore cost-effectiveness. However, there is a lack of robust and randomized control trial evidence demonstrating the increased efficacy of portable over inpatient NPWT, thus considering the homecare setting. As such, the development of portable NPWT is an encouraging innovation in wound care technology and extending the benefits to the homecare setting is both possible and potentially more beneficial.

The application of such technology at a territorial level, with the consequent definition of all the potential advantages, will be a topic for further research, thus being consistent with pillar six of the above mentioned NRRP.

Despite the successful results achieved, a key limitation of the study would be the small sample of healthcare professionals involved in the qualitative analysis. Further analyses may be conducted in the future to validate all the findings.

## 5. Conclusions

This study provides helpful evidence-based information to policymakers through the examination of the relative values of intervention, thus supporting the overall hospital and institutional decision-making process to define the appropriate areas of investments, leading to the achievement of not only higher clinical outcomes, but also important social, economic, and organizational advantages.

## Figures and Tables

**Table 1:** Endpoints from the literature.

Study	No. of Patients Enrolled	Technologies Being Compared	Surgical Site Infection (SSI)	Reduction of Hospital Stay (Days)	Re-Hospitalization	Re-Operation	Type of Study
Pleger SP. et al. 2018 [14]	100 patients	NPWT vs. traditional dressing	SSI rate considering a 30-day time horizon: 8.3% vs. 21.1%, *p* = 0.023	12.8 days vs. 13 days, *p* > 0,05	Not available	Re-operation rate considering a 30-day time-horizon: 1.7 % vs. 14.1% (*p* = 0.022)	RCT
Gombert A. et al. 2018 [12]	204 patients	NPWT vs. traditional dressing	13.2% vs. 33.3%, *p* = 0.0015	Not significant (both 8 days)	Not available	Not significant (5 vs. 6)	RCT
Kwon J. et al. 2018 [11]	119 patients	NPWT vs. traditional dressing	SSI rate considering a 30-day time horizon: 30 g. 10.1% vs. 21.6% all, *p* = 0.001	9.1 days vs. 10 days, *p* > 0,05	Re-hospitalization rate considering a 30-day time-horizon: 6.8% vs. 16.7%, *p* = 0,04	Re-operation rate considering a 30-day time-horizon: 8.5% vs. 18.3%, *p* = 0.05	RCT
Matatov T.et al 2013 [13]	115 patients	NPWT vs. traditional dressing	6% (III°) vs. 30% (16% I°-11%II°-19%III°) *p* = 0.0011	Not available	Not available	Not available	Observational Study
Lee K. et al. 2017 [10]	102 patients	NPWT vs. traditional dressing	SSI rate considering a 30-day time horizon: 11.3% vs. 18.4 % *p* = 0.24 SSI rate considering a 90-day time horizon: 13.2% vs. 22.4% *p* = 0.26	Average length: 6.4 days NPWT vs. 8.9 days with traditional dressing, *p* = 0.02	Not significant *p* = 0.93	Not significant *p* = 0.53	RCT

**Table 2:** Economic valuation of the two patients’ pathways.

	Surgery		Hospitalization		Outpatient’ Follow Up		Total	
	Traditional	NPWT	Traditional	NPWT	Traditional	NPWT	Traditional	NPWT
Human resources	EUR 1196	EUR 1196	EUR 655	EUR 473	EUR 22	EUR 13	EUR 1874	EUR 1683
Equipment and facilities	EUR 80	EUR 80	EUR 0	EUR 0	EUR 0	EUR 0	EUR 80	EUR 80
Consumables	EUR 959	EUR 1265	EUR 11	EUR 1.40	EUR 1.40	EUR 0	EUR 972	EUR 1266
Other	EUR 1351	EUR 1351	EUR 5400	EUR 3900	EUR 89	EUR 51	EUR 6840	EUR 5302
General Cost	EUR 717	EUR 778	EUR 1213	EUR 875	EUR 23	EUR 13	EUR 1953	EUR 1666
Total per phase and per entire process	EUR 4304	EUR 4670	EUR 7280	EUR 5250	EUR 135	EUR 77	EUR 11,719	EUR 9997

**Table 3:** Budget impact analysis.

	Population Traditional Medication	Population NPWT	Pathway Traditional Medication	Pathway NPWT	Total	Savings %
Base Scenario	100%	0%	EUR 9,058,814	EUR 0	EUR 9,058,814	
Scenario 1	80%	20%	EUR 7,461,150	EUR 1,802,681	EUR 9,263,831	2.26%
Scenario 2	50%	50%	EUR 5,064,655	EUR 3,890,317	EUR 8,954,972	−1.15%
Scenario 3	20%	80%	EUR 2,668,160	EUR 5,977,953	EUR 8,646,112	−4.56%
Scenario 4	0%	100%	EUR 0	EUR 7,369,710	EUR 7,369,710	−18.65%

**Table 4:** Healthcare professionals’ perceptions.

Safety Profile	Traditional Dressing	NPWT
Perception about the development of severe adverse events for patients	−1	1
Perception about the development of moderate adverse events for patients	0.33	1.67
Invasiveness of the procedure	0.67	0
General safety of the technology	0.67	1
Technology’s tolerability	0	0.67
Perception about the development of any procedural risks for the healthcare professionals involved	0	0.33
Perception about the development of any risks for the caregivers	−0.33	0.67
Environmental risk	0	0
Level of criticality in handling the surgical incision	−0.33	1
Ease of use of the technology by the operator	−0.33	0.67
Perception of the complexity of the procedure	0.33	1
Impact of new technology on disease progression	0.33	1.33
***Average value for safety profile***	***0.03***	***0.78***
**Effectiveness Profile**	**Traditional Dressing**	**NPWT**
Improvement in patient-reported outcomes	0.67	2
Impact of the technology on mortality rates	0.67	2
Impact of technology on the ability to prevent surgical site infections	0.67	2.33
Impact of technology on the occurrence of debridement events	0,33	2.33
***Average value for effectiveness profile***	***0.58***	***2.17***
**Equity Profile**	**Traditional Dressing**	**NPWT**
Accessibility of technology in the territory	0.66	−0.33
Accessibility of the technology to persons of a legally protected status	0.66	−0.33
Impact of the drug on waiting lists	0	−0.33
Ability of the technology to generate health migration phenomena in case of use	0	0
Existence of factors that could facilitate a group or certain types of patients to benefit from the technology	0	−0.33
***Average value for equity profile***	***0.26***	***−0.26***
**Social Profile**	**Traditional Dressing**	**NPWT**
Ability of the technology to protect the patients’ autonomy	0.33	1.67
Ability of the technology to protect human rights	1	1
Ability of the technology to protect the patients’ integrity	0	1.33
Ability of the technology to protect the patients’ religion	0	0
Impact of the technology on social costs	1	1.33
Patients and citizens can have a good level of understanding of the drug	0.67	1
Impact of the technology on the length of stay	0.33	1.67
Impact of the technology on the patient’s perceived quality of life	0.33	1.33
Impact of the technology on the care givers’ quality of life	0	1.33
Impact of the technology on patients’ perceived pain	0.33	1.33
***Average value for social profile***	***0.39***	***1.19***
**Legal Profile**	**Traditional Dressing**	**NPWT**
Authorization level	0.33	0.33
Need for inclusion of the drug in a national/European register	0.33	0.33
Safety requirement	1.33	1.33
Intellectual property rights infringement	0.00	0
Need to regulate the acquisition of the technology	0.33	0.33
Legislation covers regulation of technology for all categories of patients who can benefit from the drug in accordance with the specific technical indications	0.00	0
***Average value for legal profile***	***0.39***	***0.39***

## Data Availability

The data that support the findings of this study are available from the corresponding author, [AV], upon reasonable request.
